# Conventional Versus Regenerative Methods for Wound Healing: A Comparative Experimental Study on a Sheep Model

**DOI:** 10.3390/medicina60111836

**Published:** 2024-11-08

**Authors:** Rossella Elia, Michele Maruccia, Pietro Giovanni Di Summa, Rodrigo Trisciuzzi, Giuditta Lovero, Gerardo Cazzato, Luca Lacitignola, Francesco Staffieri, Alberto Maria Crovace

**Affiliations:** 1 Unit of Plastic and Reconstructive Surgery, Department of Precision and Regenerative Medicine and Jonic Area, University of Bari, 11, Piazza Giulio Cesare, 70124 Bari, Italy; 2 Department of Plastic and Hand Surgery, Centre Hospitalier Universitaire Vaudois (CHUV), University of Lausanne (UNIL), Rue du Bugnon 46, 1011 Lausanne, Switzerland; 3 Department of Precision-Regenerative Medicine and Jonic Area (DiMePRe-J), Section of Veterinary Medicine, University of Bari “Aldo Moro”, 70124 Bari, Italy; 4 Section of Molecular Pathology, Department of Precision and Regenerative Medicine and Ionian Area (DiMePRe-J), University of Bari Aldo Moro, 70124 Bari, Italy; 5 Department of Veterinary Medicine, University of Sassari, 07100 Sassari, Italy

**Keywords:** wound, wound bed preparation, dermal matrix, micrograft

## Abstract

*Background and Objectives*: Wound healing is a complex process involving cellular, anatomical, and functional repair, often hindered in chronic wounds associated with diseases like diabetes and vascular disorders. This study investigated the efficacy of conventional and regenerative wound healing approaches in a sheep surgical wound model. *Materials and Methods*: Six female Bergamasca sheep underwent five full-thickness skin lesions treated with various methods: sterile gauze (control), chlorhexidine, sodium hypochlorite, micronized dermis system application, and dermal matrix. Wound healing progression was monitored over 42 days through wound dimension measurements, exudate analysis, and histopathological evaluations. *Results*: The results indicated that all wounds healed completely by day 42, with significant reductions in wound size and exudate over time. Notably, Micronized dermis system application and dermal matrix treatments showed a faster evolution in exudate characteristics and improved collagen reorganization compared to other treatments. Histological analysis revealed earlier neovascularization and better reconstitution of hair follicles in these groups. Despite the lack of significant differences in healing time, both regenerative approaches enhanced wound healing phases, contributing to exudate control, angiogenesis promotion, and reduced scar formation. *Conclusions*: The findings suggest that while micronized dermis system application and dermal matrix do not accelerate acute wound healing compared to conventional methods, they offer potential benefits in managing exudate and improving tissue regeneration, warranting further investigation in chronic wound scenarios.

## 1. Introduction

A wound is defined as a disruption of the cellular, anatomical, and functional continuity of a living tissue and may be caused by physical, chemical, thermal, microbial, or immunological insults [1]. The social and economic burden of wounds worldwide is a consequence of their increasing rate, especially in the aging population [2]. Moreover, in addition to a high number of acute wounds, there is also a large number of chronic ones, namely, those that do not progress through the healing process in a timely manner [3], usually associated with an underlined disease (vascular, diabetic, and pression ulcers) [4]. It is estimated that more than six million people suffer from chronic wounds every year in the United States, and the incidence is estimated to grow by 8%, due to the aging of the population and the spread of diseases such as diabetes, obesity, and vascular diseases [5].

Acute wound healing is a well-regulated process consisting of partially overlapping phases that are determined by interacting events on a molecular, cellular, and extracellular matrix level that ends with wound closure within days or weeks. Conversely, when the healing process does not occur, it is the consequence of an intricate interplay of local tissue factors, systemic comorbidities, and environmental influences. Persistent ischemia, inflammation, and microbial colonization are key drivers in the perpetuation of the wound-healing cascade, often leading to a prolonged inflammatory phase and impaired tissue regeneration [6,7,8,9].

Despite the intense investigation, numerous strategies and advancements in skin wound healing, management of large wounds and chronic wounds still remains an unmet therapeutic area due to difficulty in its assessment and wound care management [10]. Therefore, the research for the development of improved and innovative strategies for skin wound healing holds profound global medical importance in the healthcare domain. Alongside “conventional” skin wound therapies based on debridement and dressing changes, more advanced “regenerative” approaches have been described, including bioactive biomaterial matrices and stem cell therapies [11,12,13,14]. Recently, an innovative technology for dermal mechanical micro-fragmentation has been developed, allowing for the harvesting of a filtered available cell pool rich in progenitor cells without any enzymatic manipulation.

The aim of the present study is to compare different conventional and regenerative approaches to enhance wound healing in a sheep surgical wound model based on clinical and histopathological analyses. The ultimate goal is to contribute to research in the field of wound healing, helping to choose the most appropriate strategy with potential application in both veterinary and human fields.

## 2. Materials and Methods

### 2.1. Experimental Models and Procedure

**Figure 1:**
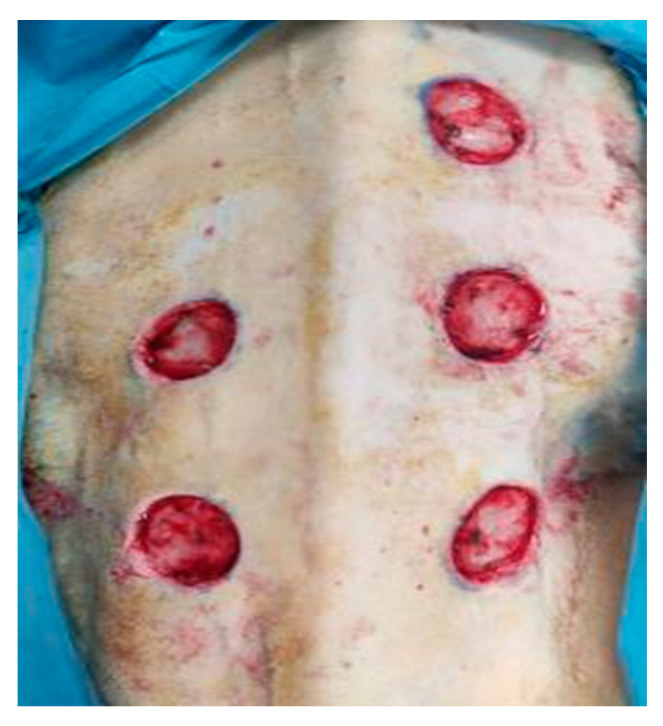
Control and experimental skin lesions produced on the backs of the sheep.

After approval by the Italian Ministry of Health (n. 324/2020-PR, 16 April 2020), and in strict accordance with the recommendations in the Guide for the Care and Use of Laboratory Animals of the National Institutes, six female Bergamasca breed sheep of similar age and weight (54 ± 6.7 kg) were used. All animals, after fasting for 24 h, were premedicated with intramuscular midazolam and buprenorphine, and subsequently, venous and arterial accesses were positioned. Following from this, general anesthesia was induced for each experimental animal using Propofol, and this was maintained with a halogenated anesthetic (Isoflurane). First, hair was removed from dorsal and lumbar regions through an electric hair clipper and refined with a manual shaver. Each animal was then placed in sternal recumbency, and 5 full thickness skin lesions (A–E) were produced on the backs of the sheep according to the protocol established by Broeckx et al. [15] (Figure 1).

During the preoperative scrub, performed alternately with chlorhexidine and povidone–iodine solution on the back of the animal, a plastic guide was used to trace the circumference of each lesion. The lesions measured four centimeters in diameter and were placed at least 4 cm distant from each other in order to make their healing process as independent as possible. The lesions were produced with a cold blade, and the intraoperative hemorrhages were stopped using electrosurgical devices.

**Figure 2:**
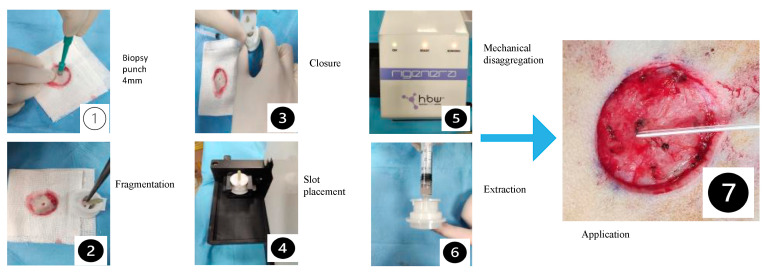
Micronized dermis system application.

The lesions were classified and treated as follows:

- Lesion A (control area). It was treated exclusively with a sterile gauze and physiological solution (wet-to-dry).
- Lesion B (experimental). It was treated with a superficial disinfection of 0.05% diluted chlorhexidine.
- Lesion C (experimental). It was treated with a 0.05% sodium hypochlorite solution.
- Lesion D (experimental). It was treated intraoperatively with the Rigenera^®^ (Human Brain Wave, Turin, Italy) system. The protocol consists of four steps: (i) collection of a small piece of skin tissue, (ii) disaggregation of tissue through the addition of sterile saline solution, (iii) collection of autologous micro-grafts obtained after the disaggregation, and (iv) injection of these micro-grafts alone into the site of injury. Specifically, the surgically excised skin as full thickness was preserved and destined for processing accordingly. A total of 1.2 mL sterile injectable physiologic solution was added through the specific connector into the Micronized dermis system application © device. The harvested tissue sample was placed on the grid inside the machine, which was connected to the Sicurdrill Device. The Sicurdrill Device was activated for 2 cycles (with each cycle lasting 1 min). The micro-graft suspension was collected with a syringe from the same hole in which the saline solution was previously injected, being diluted to 2 mL of solution. One-half of the obtained micrograft solution was injected in the wound bed, and the remaining half into the wound edges (4 mm deep) (Figure 2).
- Lesion E (experimental). It was treated intraoperatively by the application of PELNAC™ (Gunze, Japan), a dermal matrix with a double fenestrated layer. It is composed of porcine atelocollagen and a layer of silicone; it is available in different sizes and types based on the surgeon’s needs and the characteristics of the lesion. The entire lesion area was covered with a single piece of the dermal substitute. A secondary medication with sterile gauze was performed.

Wound cleaning and bandages changes were performed on all wounds daily.

### 2.2. Outcome Evaluation

The primary outcome of the study was the evaluation of the time required for the lesions to heal while the scar quality at the end of the process was assessed as a secondary outcome.

**Figure 3:**
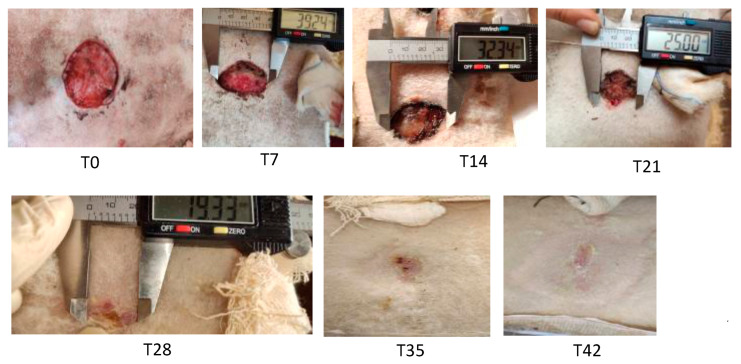
Progressive wound contraction and healing over time. Wound diameter was evaluated at each time point with a caliper.

Wounds were photographed every 7 days (Figure 3) and subjected to a blind evaluation by a team of independent researchers. Wound dimensions were recorded using an electronic caliper at T7 (7 days), T14 (14 days), T21 (21 days), T28 (28 days), and T42 (42 days). The amount of exudate, its color, and its character, together with hydration status and appearance of the dressing gauze, were evaluated and rated according to the Hadley rating scale [16] (Table 1).

At two time points (T14 and T42), one sample from each lesion was obtained by 6 mm punch biopsy using sedation and analgesic drug administration. The samples were fixed in 10% neutral-buffered formalin and routinely processed for light microscopy. Representative sections were stained with hematoxylin and eosin, and they were microscopically evaluated by a single board-certified dermatopathologist blinded to the grouping of the biopsy. The following characteristics were evaluated for all samples: the presence of dermal and subcutaneous infiltrates, (immature) granulation tissue, undifferentiated mesenchymal tissue, and the development of adnexa. These characteristics were scored using a 0 to 4 scale (0 = absence, 1 = presence, 2 = small amount, 3 = moderate amount, 4 = abundant amount). Data were presented as the relative frequencies of the assigned values and calculated for each sheep and for each parameter.

### 2.3. Statistical Analysis

The data collected were entered into Office Excel software and analyzed using Stata MP16 software version 18. Continuous variables were expressed as mean ± standard deviation and range. Assessments were normally distributed according to the Shapiro–Wilk test. Repeated measures analysis of variance (ANOVA) with a Bonferroni post hoc test was performed to analyze the time effect, treatment effect, and time–treatment interaction effect for the wound dimension, presence of exudate, color of exudate, type of exudate, gauze appearance, and hydration. A *p*-value < 0.05 was considered statistically significant.

## 3. Results

### 3.1. Wound Dimension

**Figure 4:**
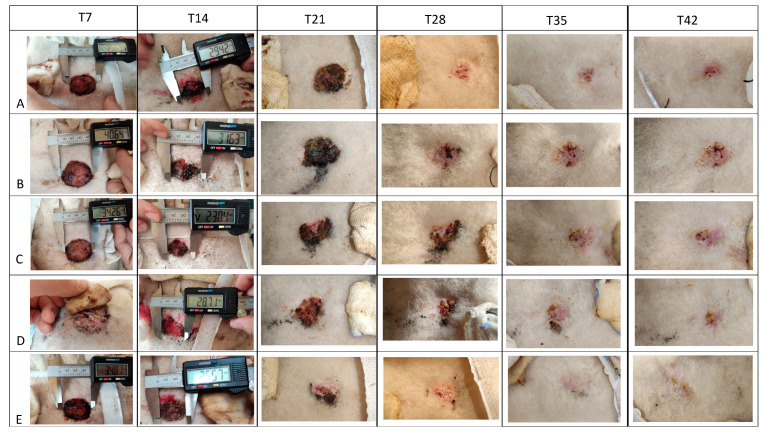
Pictures refer to sheep n.1 and show the clinical appearance of the wounds belonging to different treatment methods (from A to E) at each time point.

All the wounds from experimental and control groups showed a fairly homogenous decrease in terms of diameter over time, with a progressive reduction in size, up to their complete closure at T42 in all groups. The comparison between mean wound diameters showed a significant difference only between the “Micronized dermis system vs. control” and “sodium hypochlorite vs. control” groups (*p* < 0.01*). A significant time effect was observed in all cases, while no significant interaction effect (group-by-time) was reported (Figure 4, Table 2 and Table 3).

### 3.2. Presence of Exudate

The exudate reduced progressively over time in all groups, with no significant treatment effect. A time effect was seen in all cases, while a significant group by time interaction was found in the cases of “dermal matrix vs. chlorhexidine” and “dermal matrix vs. sodium hypochlorite” (Table 4 and Table 5).

### 3.3. Color of Exudate

In relation to the “color of the exudate” parameter, all groups showed a first increase in the recorded values between T0 and T7, followed by a decrease, progressing from a green exudate to a clear one until its disappearance at the end of the healing process. The group treated with the Micronized dermis system protocol reached the value of 0 at 28 days, while the group treated with the dermal matrix did the same at 35 days. The comparison between groups showed a significant difference only in case of “chlorhexidine vs. control”. A time effect was observed in all cases except for the “dermal matrix vs. control” comparison. In the analysis of the interaction between times and groups, statistically significant results were obtained for the comparison “dermal matrix vs. control”, “micronized dermis system vs. control”, “chlorhexidine vs. control”, “dermal matrix vs. chlorhexidine”, “dermal matrix vs. sodium hypochlorite”, and “dermal matrix vs. micronized dermis system” (Table 6 and Table 7).

### 3.4. Type of Exudate

The exudate evolves from a purulent aspect to a progressively serum-ematic and serum form until disappearing. A faster evolution was noted in the group treated with the Micronized dermis system protocol, followed by the one treated with the dermal matrix. By comparing the average values of the parameter “type of exudate” between the groups, statistically significant results were obtained in the case of the comparison “chlorhexidine vs. control” and “dermal matrix. vs. chlorhexidine”. A time effect was observed in all cases, while a significant group by time interaction was found in the case of “dermal matrix. vs. control”, “Micronized dermis system vs. control”, “chlorhexidine vs. control”, “dermal matrix. vs. chlorhexidine”, “dermal matrix. vs. sodium hypochlorite”, and “dermal matrix. vs. Micronized dermis system” (Table 8 and Table 9).

### 3.5. Appearance of the Gauze

All the groups showed a progressive reduction in terms of humidity degree, reaching a state of dry gauze by the end of the observation time with no significant treatment effect. A time effect was seen in all cases, with no significant group by time interaction effect (Table 10 and Table 11).

### 3.6. Hydration of the Gauze

All the groups showed a progressive reduction in terms of wound hydration with time, and no statistical significance was observed after the comparison between groups. A time effect was found in all cases, with no group by time interaction (Table 12 and Table 13).

### 3.7. Histological Results

Histopathological examination showed no significant differences between experimental and control groups at T14 in terms of immature granulation tissue, except for the presence of numerous fibroblasts observed in wounds treated with dermal matrix and the Micronized dermis system, which piled up between condensed bundles of collagen, and while loose, unorganized collagen was observed in the remaining groups.

Specifically, dermal and subcutaneous inflammation were seen in moderate amounts in all groups (mean score of 3.2 ± 0.7), with no significant differences among groups. Immature granulation tissue was abundant in all cases (mean score of 3.8 ± 0.2), and no cutaneous adnexa were observed.

**Figure 5:**
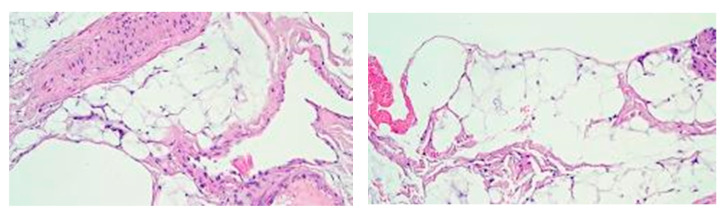
Histological photomicrograph of the healed tissue at T42 (sheep n.1). The image on the left belongs to the dermal matrix, while the image on the right belongs to the control area. The almost complete absence of dermal and subcutaneous infiltrate and granulation tissue could be observed (HE original magnification 20×).

**Figure 6:**
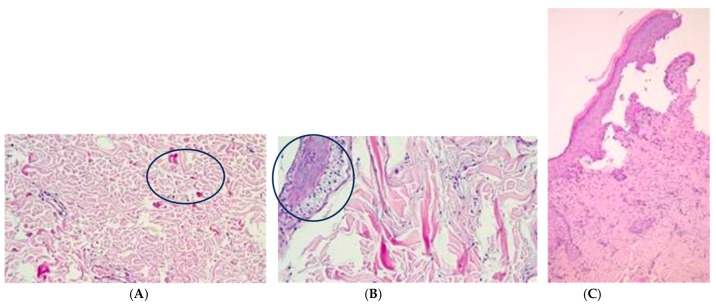
(**A**–**C**) Histological photomicrograph taken at T42 showing in (**A**) the reorganization of the collagen fibers in the DERMAL MATRIX group (HE original magnification 20× example in the black circle). (**B**) Another histological photomicrograph of the same DERMAL MATRIX group showing the reappearance of cutaneous adnexa (left, black circle) and the reorganization of the collagen fibers in the middle of the picture (HE original magnification 20×). (**C**) Histological photomicrograph showing the keratinization of the epidermis (HE original magnification 4×).

At T42, inflammation was completely absent at the subcutaneous layer. Dermal inflammation was still present in a small amount in the control area, as well as in the “conventional” treated areas (mean scores of 0.4 ± 0.2). Granulation tissue was absent from all the wounds in parallel with the completion of the wound healing process in all groups (Figure 5). The samples belonging to the dermal matrix and micronized dermis system group at T42 showed reorganization of collagen with keratinization of the skin (Figure 6).

**Figure 7:**
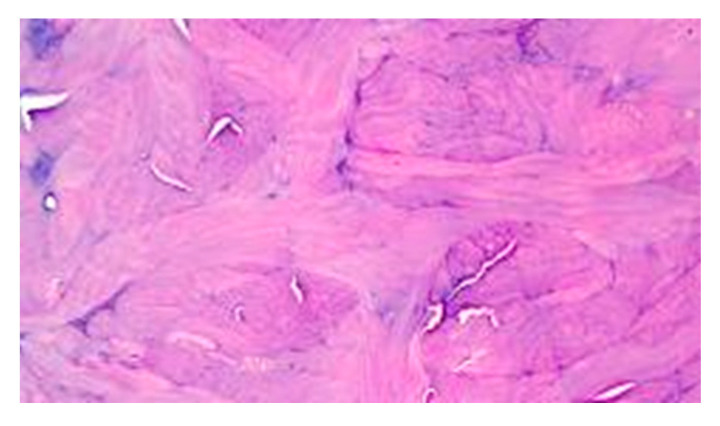
Histological photomicrograph showing poor scarring with advanced fibrosis in the control area (HE original magnification 20×).

Undifferentiated mesenchymal tissue and cutaneous adnexa were present in all the samples but in different densities among groups (mean scores of 1.1 ± 0.4 for lesions A-B-C and 2.3 ± 0.8 for lesions D-E). Interestingly, hair follicles and sebaceous and apocrine glands were present in all samples, but the cutaneous adnexa observed in the samples belonging to dermal matrix and Micronized dermis system appeared more mature and denser (Figure 6B) compared to the remaining group, where poor scarring and advanced fibrosis were observed (Figure 7).

## 4. Discussion

Experimental wound healing models have been developed over many decades in an attempt to understand the tissue repair process and test new treatment protocols. In vivo models remain the most predictive models for studying wound healing, allowing a realistic representation of the wound environment including various cell types, environmental cues, and paracrine interactions. Acute models are easier to set up, while animal models being used in this field of research have failed to recapitulate the clinical features of a chronic wound [4].

The present study used sheep as an experimental model for several reasons. Sheep share many anatomical and physiological similarities with humans, with comparable brain size and body weight [17]. Further, their housing is relatively cheap; it is easy to gain a peripheral venous and arterial access; they are less neurologically developed than carnivores and equines; and they have a sufficient dorsal surface space for the creation of different experimental lesions on the same subject, reinforcing the statistical standardization value of the study [18]. The number of sheep was chosen based on sample size calculation and was defined according to the “3Rs” principle (replacement–reduction–refinement).

The authors’ work is not isolated in the research panorama using a sheep model for skin wound research. Badis and Omar [19] showed how the topical administration of platelet-rich plasma improved the skin healing process by promoting epithelialization after three weeks of wounding. A similar model was used recently by Martinello and colleagues [20] to compare secondary intention wound healing after treatment with topical allogeneic mesenchymal stem cells. Nevertheless, to the best of our knowledge, this is the first prospective study comparing conventional approaches and the methods of dermal matrix and Micronized dermis system for wound healing.

Reading the results in a critical manner, all treatment strategies were effective in determining wound healing. There was no difference in terms of time required to heal between the experimental approaches, either conventional or regenerative and the control, which was the primary endpoint of the study. Arguably, in the acute wound healing model, the absence of known risk factors for the impaired healing process and the adequate hygiene condition put all the injuries in the optimal environment to heal. The attention to the hygienic conditions certainly also contributed to the evidence that no frankly purulent exudate nor infection was observed in any case. This is the reason why a study of the microflora was not conducted.

However, the analysis of the data related to the individual aspects of the wound bed does reveal some interesting findings. The reduction in wound size occurred in parallel with the progressive reduction in exudate, and this was an intuitive finding. Similarly, the character of the exudate varied over time, from an initial corpuscular phase (typical of the inflammatory process) to a clearer appearance. “Time” was a determinant factor in all cases for the reduction in size of the wounds and for the exudate and hydration state modifications. Still, the interaction analysis showed a significant difference only for the dermal matrix and micronized dermis system vs. control in terms of color and type of exudate, meaning that the type of treatment chosen influenced the variations of the presence and characteristics of exudate at different time points. Now, in the model described, healing was achieved in all cases, but the positive group by time interaction related to the exudate features for the dermal matrix and micronized dermis system protocol may suggest a useful application of both approaches for the treatment of those clinical scenarios (wound bed preparation) where a better control over the exudate needed to be achieved [21,22,23,24]. As a matter of fact, moisture balance is part of the T.I.M.E. (“Tissue, Inflammation/Infection, Moisture, Edge/Epithelialization”) protocol, which was introduced with the aim of promoting the acceleration of the wound repair process [25,26,27,28].

The histological analysis of tissue samples shows that all the treatments led to a complete epithelization at T42. Nevertheless, at the same time point, the newly formed tissue presented some differences among the different protocols. Both the lesions treated with dermal matrix and the micronized dermis system protocol allowed for a good epithelialization, with earlier neovascularization followed by a more elaborate collagen reorganization, whereas a proper scar tissue was observed in the remnant cases. Further, concerning the reconstruction of hair follicles, the results of this present study showed that there was some reconstitution of the dermal papilla cells in the samples treated with dermal matrix and micronized dermis system protocol (Figure 3, Figure 4 and Figure 5). This finding corroborates the idea that both strategies could be considered regenerative approaches for wound healing. Several studies, both in vitro and vivo, have demonstrated that the presence of acellular dermal matrix augments and modulates the wound healing process to its advantage by simultaneously increasing the invasion of appropriate cellular constituents to facilitate expeditious healing and accelerate angiogenesis [23,29,30,31,32]. Analogously, in the clinic, Riccio et al. [33] identified progenitor cells able to initiate regeneration and enhance wound healing in the micronized dermis. Moreover, they demonstrated that the micronized dermis system was effective in stimulating skin regeneration while reducing scarring in the reconstruction of full-thickness posttraumatic skin defects of the limbs.

Evaluating clinical and histological findings as a whole, the idea is that both the dermal matrix and the micronized dermis system may regulate and improve the phases of wound healing, contributing to the reduction of inflammation, promoting angiogenesis, and in the end attenuating scar formation.

The small sample size and the acute wound setting are obvious limitations of the present study. Still, the results are of scientific interest for the comparison of several treatment protocols under standard conditions.

## 5. Conclusions

The application of a dermal matrix or the use of the micronized dermis system do not enhance the healing of acute, full-thickness trunk wounds in sheep as for the protocol used in this study, compared to conventional treatments or even compared to no topical disinfectant application at all. However, both approaches did appear to improve the phases of wound healing, contributing to the exudate control, promoting angiogenesis, and in the end attenuating scar formation. Further investigations in the mid-to-late repair stage of healing are indicated.

## Figures and Tables

**Table 1:** Wound evaluation.

Parameter	Score
Presence of exudate	1 absent 2 small 3 moderate 4 abundant
Color of exudate	1 clear 2 pink/red 3 brown 4 yellow 5 green
Character of exudate	1 serous 2 serosanguineous 3 sanguineous 4 purulent + 5 purulent ++ 6 purulent +++
Gauze	1 dry/clean 2 dry/stained 3 moist 4 wet
Hydration	1 normal 2 maceration + 3 maceration ++ 4 desiccation + 5 desiccation ++

+ mild; ++ moderate; +++ severe.

**Table 2:** Wound diameter by group and time.

Group	T0	T7	T14	T21	T28	T35	T42
Control	40.0 ± 0.0 (40–40)	29.8 ± 4.1 (23.4–34.3)	25.6 ± 4.9 (17.3–29.9)	17.6 ± 4.7 (9.7–23.2)	4.2 ± 6.8 (0.0–16.1)	0	0
Sodium hypochlorite	400 ± 0.0 (40–40)	37.3 ± 4.5 (32.1–45.0)	29.5 ± 1.9 (26.6–32.2)	22.3 ± 3.0 (18.9–26.1)	7.0 ± 8.0 (0.0–18.3)	0	0
Chlorhexidine	40.0 ± 0.0 (40–40)	36.3 ± 5.2 (28.9–40.6)	27.0 ± 4.6 (18.7–31.6)	19.1 ± 10.1 (0.0–25.7)	9.3 ± 10.6 (0.0–23.0)	0	0
Dermal matrix	40.0 ± 0.0 (40–40)	*	26.5 ± 4.0 (22.2–32.7)	20.0 ± 2.6 (16.7–23.7)	3.8 ± 6.2 (0.0–14.3)	0	0
Micronized dermis system	40.0 ± 0.0 (40–40)	36.0 ± 4.0 (29.3–40.8)	27.4 ± 3.0 (24.2–31.2)	22.2 ± 2.8 (18.9–25.4)	7.9 ± 9.0 (0.0–19.7)	0	0
Mean ± SD	40.0 ± 0.0 (40–40)	34.9 ± 5.1 (23.4–45.0)	27.2 ± 3.8 (17.3–32.7)	20.2 ± 5.4 (0.0–26.1)	6.4 ± 8.0 (0.0–23.0)	0	0

All measures are expressed in cm (range). * No evaluation was performed at T7 in the case of dermal matrix.

**Table 3:** Comparison of wound diameters by group, time, and group by time (ANOVA test).

	Group	Time	Group by Time
Dermal matrix vs. control	0.585	<0.0001	0.937
Micronized dermis vs. control	0.008	<0.0001	0.348
Chlorhexidine vs. control	0.189	<0.0001	0.495
Sodium hypochlorite vs. control	0.008	<0.0001	0.144
Dermal matrix vs. chlorhexidine	0.582	<0.0001	0.596
Micronized dermis vs. chlorhexidine	0.859	<0.0001	0.967
Sodium hypochlorite vs. chlorhexidine	0.674	<0.0001	0.854
Dermal matrix vs. sodium hypochlorite	0.084	<0.0001	0.709
Micronized dermis vs. sodium hypochlorite	0.524	<0.0001	0.979
Dermal matrix vs. micronized dermis	0.062	<0.0001	0.726

**Table 4:** Presence of exudate by time.

Group	T0	T7	T14	T21	T28	T35	T42
Control	1.8 ± 0.8 (1–3)	1.3 ± 0.5 (1–2)	1.0 ± 0.0 (1–1)	1.0 ± 0.0 (1–1)	1.2 ± 0.4 (1–2)	1.0 ± 0.0 (1–1)	1.0 ± 0.0 (1–1)
Sodium hypochlorite	1.8 ± 0.8 (1–3)	2.2 ± 0.8 (1–3)	1.0 ± 0.0 (1–1)	1.0 ± 0.0 (1–1)	1.0 ± 0.0 (1–1)	1.0 ± 0.0 (1–1)	1.0 ± 0.0 (1–1)
Chlorhexidine	2.0 ± 0.9 (1–3)	2.2 ± 0.4 (2–3)	1.0 ± 0.0 (1–1)	1.0 ± 0.0 (1–1)	1.0 ± 0.0 (1–1)	1.0 ± 0.0 (1–1)	1.0 ± 0.0 (1–1)
Dermal matrix ^®^	1.3 ± 0.5 (1–2)	1.7 ± 0.5 (1–2)	1.5 ± 0.5 (1–2)	1.2 ± 0.4 (1–2)	1.2 ± 0.4 (1–2)	1.0 ± 0.0 (1–1)	1.0 ± 0.0 (1–1)
Micronized dermis system application ©	1.8 ± 0.8 (1–3)	2.2 ± 0.4 (2–3)	1.2 ± 0.4 (1–2)	1.0 ± 0.0 (1–1)	1.0 ± 0.0 (1–1)	1.0 ± 0.0 (1–1)	1.0 ± 0.0 (1–1)
Mean ± SD	1.8 ± 0.7 (1–3)	1.9 ± 0.6 (1–3)	1.1 ± 0.3 (1–2)	1.0 ± 0.2 (1–2)	1.1 ± 0.3 (1–2)	1.0 ± 0.0 (1–1)	1.0 ± 0.0 (1–1)

**Table 5:** Comparison of exudate presence by group, time, and group by time (ANOVA test).

	Group	Time	Group by Time
Dermal matrix vs. control	0.574	<0.0001	0.13
Micronized dermis vs. control	0.257	<0.0001	0.066
Chlorhexidine vs. control	0.196	<0.0001	0.085
Sodium hypochlorite vs. control	0.358	<0.0001	0.092
Dermal matrix vs. chlorhexidine	0.651	<0.0001	0.019
Micronized dermis vs. chlorhexidine	0.971	<0.0001	0.983
Sodium hypochlorite vs. chlorhexidine	0.781	<0.0001	0.999
Dermal matrix vs. sodium hypochlorite	0.831	<0.0001	0.051
Micronized dermis vs. sodium hypochlorite	0.791	<0.0001	0.999
Dermal matrix vs. micronized dermis	0.670	<0.0001	0.08

**Table 6:** Color of exudate by time.

Group	T0	T7	T14	T21	T28	T35	T42
Control	1.0 ± 1.1 (0–2)	0.7 ± 1.0 (0–2)	0	0.2 ± 0.4 (0–1)	0.3 ± 0.8 (0–2)	0	0
Sodium hypochlorite	1.2 ± 1.0 (0–2)	1.5 ± 1.0 (0–3)	0	0.2 ± 0.4 (0–1)	0	0	0
Chlorhexidine	1.3 ± 1.0 (0–2)	2.3 ± 0.5 (2–3)	0	0.2 ± 0.4 (0–1)	0	0	0
Dermal matrix^®^	0.2 ± 0.4 (0–1)	0.7 ± 0.5 (0–1)	1.2 ± 1.0 (0–2)	0.3 ± 0.8 (0–2)	0.3 ± 0.8 (0–2)	0	0
Micronized dermis system application ©	1.2 ± 1.0 (0–2)	2.0 ± 0.6 (1–3)	0.2 ± 0.4 (0–1)	0.2 ± 0.4 (0–1)	0	0	0
Mean ± SD	1.0 ± 1.0 (0–2)	1.4 ± 1.0 (0–3)	0.3 ± 0.6 (0–2)	0.2 ± 0.5 (0–2)	0.1 ± 0.5 (0–2)	0	0

**Table 7:** Comparison of color of exudate by group, time, and group by time (ANOVA test).

	Group	Time	Group by Time
Dermal matrix vs. control	0.682	0.138	0.028
Micronized dermis vs. control	0.153	<0.0001	0.047
Chlorhexidine vs. control	0.033	<0.0001	0.007
Sodium hypochlorite vs. control	0.47	<0.0001	0.426
Dermal matrix vs. chlorhexidine	0.325	<0.0001	<0.0001
Micronized dermis vs. chlorhexidine	0.679	<0.0001	0.884
Sodium hypochlorite vs. chlorhexidine	0.245	<0.0001	0.381
Dermal matrix vs. sodium hypochlorite	0.898	<0.0001	0.0
Micronized dermis vs. sodium hypochlorite	0.508	<0.0001	0.840
Dermal matrix vs. micronized dermis	0.52	<0.0001	<0.0001

**Table 8:** Type of exudate by time.

Group	T0	T7	T14	T21	T28	T35	T42
Control	1.3 ± 1.0 (0–2)	0.8 ± 1.1 (0–2)	0	0	0	0	0
Sodium hypochlorite	1.2 ± 1.0 (0–2)	1.5–1.0 (0–3)	0	0	0	0	0
Chlorhexidine	1.6 ± 0.9 (0–2)	2.5 ± 0.8 (2–4)	0	0	0	0	0
Dermal matrix ^®^	0.2 ± 0.4 (0–1)	0.7 ± 0.5 (0–1)	1.2 ± 1.0 (0–2)	0.2 ± 0.4 (0–1)	0	0	0
Micronized dermis system application ©	1.2 ± 1.0 (0–2)	2.0 ± 0.6 (1–3)	0.2 ± 0.4 (0–1)	0	0	0	0
Mean ± SD	1.1 ± 1.0 (0–2)	1.5 ± 1.1 (0–4)	0.3 ± 0.6 (0–2)	0.1 ± 0.2 (0–1)	0	0	0

**Table 9:** Comparison of type of exudate by group, time, and group by time (ANOVA test).

	Group	Time	Group by Time
Dermal matrix vs. control	0.99	<0.0001	0.0
Micronized dermis vs. control	0.183	<0.0001	0.051
Chlorhexidine vs. control	0.028	<0.0001	0.001
Sodium hypochlorite vs. control	0.548	<0.0001	0.6660
Dermal matrix vs. chlorhexidine	0.052	<0.0001	<0.0001
Micronized dermis vs. chlorhexidine	0.360	<0.0001	0.453
Sodium hypochlorite vs. chlorhexidine	0.106	<0.0001	0.103
Dermal matrix vs. sodium hypochlorite	0.604	<0.0001	<0.0001
Micronized dermis vs. sodium hypochlorite	0.461	<0.0001	0.852
Dermal matrix vs. micronized dermis	0.24	<0.0001	<0.0001

**Table 10:** Gauze appearance by group and time.

Group	T0	T7	T14	T21	T28	T35	T42
Control	1.5 ± 1.7 (1–3)	1.2 ± 0.4 (1–2)	1	1.3 ± 0.8 (1–3)	1.2 ± 0.4 (1–2)	1	1
Sodium hypochlorite	1.5 ± 1.7 (1–3)	1–1.4 (1–3)	1	1.3 ± 0.8 (1–3)	1	1	1
Chlorhexidine	1.5 ± 1.7 (1–3)	1.2 ± 1.3 (1–3)	1	1.3 ± 0.8 (1–3)	1	1	1
Dermal matrix ^®^	1 ± 1.7 (1–3)	1.8 ± 1.5 (1–3)	1.3 ± 0.8 (1–2)	1.3 ± 0.8 (1–3)	1.2 ± 0.4 (1–2)	1	1
Micronized dermis system application ©	1.5 ± 1.7 (1–3)	1.3 ± 1.2 (1–2)	1	1	1	1	1
Mean ± SD	1.4 ± 1.5 (1–3)	1.8 ± 1.2 (1–3)	1.1 ± 0.4 (1–2)	1.3 ± 0.7 (1–2)	1.1 ± 0.3 (1–2)	1	1

**Table 11:** Comparison of gauze appearance by group, time, and group by time (ANOVA test).

	Group	Time	Group by Time
Dermal matrix vs. control	0.815	0.001	0.825
Micronized dermis vs. control	0.0635	<0.0001	0.473
Chlorhexidine vs. control	0.576	<0.0001	0.456
Sodium hypochlorite vs. control	0.71	0	0.769
Dermal matrix vs. chlorhexidine	0.845	0.001	0.873
Micronized dermis vs. chlorhexidine	0.837	<0.0001	0.999
Sodium hypochlorite vs. chlorhexidine	0.855	<0.0001	1
Dermal matrix vs. sodium hypochlorite	0.959	0.002	0.958
Micronized dermis vs. sodium hypochlorite	0.991	<0.0001	0.995
Dermal matrix vs. micronized dermis	0.949	0	0.818

**Table 12:** Gauze hydration by group and time.

Group	T0	T7	T14	T21	T28	T35	T42
Control	1	1	1	1	1	1	1
Sodium hypochlorite	1	1.2 ± 0.4	1	1	1	1	1
Chlorhexidine	1.2 ± 0.4 (1–2)	1	1	1	1	1	1
Dermal matrix ^®^	1	1	1	1	1	1	1
Micronized dermis system application ©	1	1	1	1	1	1	1
Mean ± SD	1.03 ± 0.18 (1–2)	1.03 ± 0.18 (1–2)	1	1	1	1	1

**Table 13:** Comparison of gauze hydration by group, time, and group by time (ANOVA test).

	Group	Time	Group by Time
Dermal matrix vs. control	1.000	<0.0001	1.000
Micronized dermis vs. control	1.000	<0.0001	1.000
Chlorhexidine vs. control	0.549	<0.0001	0.921
Sodium hypochlorite vs. control	0.549	<0.0001	0.921
Dermal matrix vs. chlorhexidine	0.549	<0.0001	0.921
Micronized dermis vs. chlorhexidine	0.549	<0.0001	0.921
Sodium hypochlorite vs. chlorhexidine	1.000	<0.0001	0.757
Dermal matrix vs. sodium hypochlorite	0.549	<0.0001	0.921
Micronized dermis vs. sodium hypochlorite	0.549	<0.0001	0.921
Dermal matrix vs. micronized dermis	1.000	<0.0001	1.000

## Data Availability

Data are available under reasonable request.
